# Intranasal immunization of pigs with porcine reproductive and respiratory syndrome virus-like particles plus 2′, 3′-cGAMP VacciGrade™ adjuvant exacerbates viremia after virus challenge

**DOI:** 10.1186/s12985-017-0746-0

**Published:** 2017-04-12

**Authors:** Alexandria Van Noort, April Nelsen, Angela E. Pillatzki, Diego G. Diel, Feng Li, Eric Nelson, Xiuqing Wang

**Affiliations:** 1 Department of Biology and Microbiology, South Dakota State University, Brookings, SD 57007 USA; 2 Department of Veterinary and Biomedical Sciences, South Dakota State University, Brookings, SD 57007 USA; 3 BioSNTR, South Dakota State University, Brookings, SD 57007 USA

**Keywords:** 2′, 3′-cGAMP vaccigrade™, PRRSV, Virus-like particles, Increased viremia, Intranasal immunization, Interferon-α

## Abstract

**Background:**

Porcine reproductive and respiratory syndrome virus (PRRSV) causes reproductive failure in pregnant sows and acute respiratory disease in young pigs. It is a leading infectious agent of swine respiratory complex, which has significant negative economic impact on the swine industry. Commercial markets currently offer both live attenuated and killed vaccines; however, increasing controversy exists about their efficacy providing complete protection. Virus-like particles (VLPs) possess many desirable features of a potent vaccine candidate and have been proven to be highly immunogenic and protective against virus infections. Here we explored the efficacy of PRRSV VLPs together with the use of a novel 2′, 3′-cGAMP VacciGrade™ adjuvant.

**Methods:**

Animals were immunized twice intranasally with phosphate buffered saline (PBS), PRRSV VLPs, or PRRSV VLPs plus 2′, 3′-cGAMP VacciGrade™ at 2 weeks apart. Animals were challenged with PRRSV-23983 at 2 weeks post the second immunization. PRRSV specific antibody response and cytokines were measured. Viremia, clinical signs, and histological lesions were evaluated.

**Results:**

PRRSV N protein specific antibody was detected in all animals at day 10 after challenge, but no significant difference was observed among the vaccinated and control groups. Surprisingly, a significantly higher viremia was observed in the VLPs and VLPs plus the adjuvant groups compared to the control group. The increased viremia is correlated with a higher interferon-α induction in the serum of the VLPs and the VLPs plus the adjuvant groups.

**Conclusions:**

Intranasal immunizations of pigs with PRRSV VLPs and VLPs plus the 2′, 3′-cGAMP VacciGrade™ adjuvant exacerbates viremia. A higher level of interferon-α production, but not interferon-γ and IL-10, is correlated with enhanced virus replication. Overall, PRRSV VLPs and PRRSV VLPs plus the adjuvant fail to provide protection against PRRSV challenge. Different dose of VLPs and alternative route of vaccination such as intramuscular injection should be explored in the future studies to fully assess the feasibility of such a vaccine platform for PRRSV control and prevention.

## Background

Despite the use of live attenuated and killed vaccines, PRRSV is still the leading cause of porcine reproductive and respiratory disease complex and results in multi-million dollar losses annually in the U.S. [13]. This is partly due to the limited efficacy of killed vaccines and the lack of cross protection of live attenuated vaccines against heterologous virus strains [12, 18, 22]. Additionally, PRRSV infection displaying persistence in the host causes grave concern about using live attenuated vaccines for the control and prevention of this disease [1, 7]. Studies also show that vaccinating boars with attenuated vaccines adversely affects the quality of sperm, and PRRSV is still detectable in semen in vaccinated boars after virulent challenge [24]. Moreover, the severe atypical PRRS complex can occur in vaccinated animals, which will continue causing tremendous economic losses [17, 30]. Another major drawback of the current vaccines includes the inability to differentiate between vaccinated and naturally infected animals. Therefore, we urgently need novel vaccine approaches to overcome the limitations of these current vaccines.

Virus-like particles (VLPs) possess many attractive and desirable features of a potent vaccine candidate and have proven effective as a vaccine strategy against human papillomavirus (HPV) infections [6]. VLPs can be manufactured easily and quickly, which offers an advantage against a highly variable virus like PRRSV. Heterologous protection can be achieved by immunizing animals with a mixture of VLPs generated using the exact genetic match of the circulating virus strains. Scientists can easily differentiate vaccinated animals from naturally infected animals by RT-PCR or the absence of nonstructural protein specific antibodies. These properties support the notion that VLPs represent one of the most promising alternative vaccine strategies for the highly variable and persistent PRRSV.

PRRSV encodes seven structural proteins, which include nucleocapsid (N), membrane (M), envelope (E), glycoproteins 2, 3, 4, and 5 (GP2, GP3, GP4, GP5). Several previous studies have reported the immunogenicity of PRRSV VLPs or chimera VLPs containing GP5 or GP5 and M proteins or the conserved protective epitopes of viral structural proteins in mice [19, 20, 26, 27]. Only one recent study described the partial protection against PRRSV in pigs immunized with PRRSV VLPs containing GP2, GP3, GP4, GP5 and M proteins [3]. More research is needed to fully explore the potential utility of VLPs in PRRSV vaccine development.

Type I interferon (IFN), the most important innate defense mechanism against virus infection, is essential to the induction of a robust adaptive immunity. Cyclic GMP-AMP, cGAMP, is an adjuvant currently being explored for its ability to increase type I interferon production. 2′, 3′ cGAMP is a cyclic dinucleotide, CDN, synthesized by cGAMP synthase (cGAS) and initiates signaling by first binding to a stimulator of IFN genes (STING) [10]. cGAS and STING are both necessary components for the 2′, 3′ cGAMP to be able to induce type 1 interferon production [25]. The IgA and IgG production in mucosal tissues after intranasal administration supports the use of this novel class of adjuvant in vaccines against respiratory diseases [15].

In this study, we generated PRRSV VLPs by using the baculovirus expression system. The immunogenicity and protective efficacy of PRRSV VLPs with or without the use of the 2′, 3′ cGAMP were evaluated in pigs.

## Methods

### Cells and recombinant baculoviruses

Sf9 insect cells (BD Biosciences) cultured in the TNM-FH Insect Cell Culture Media (BD Biosciences) were used in the generation of recombinant baculoviruses. TriEx™ Sf9 cells, serum-free adapted Sf9 derived cells, cultured in Novagen TriEx™ Insect Cell Media (Novagen, San Diego, CA) were used in the generation of VLPs by co-infection of cells with four separate recombinant baculoviruses. Recombinant baculoviruses were constructed using the FlashBac™ expression system (Oxford Expression Technologies). Briefly, PRRSV-23983 M, N, GP5, and E genes were first cloned into the transfer vector pOET1 [29]. After transfection of Sf9 cells with the transfer vector and the Flashbac baculovirus DNA using baculoFECTIN transfection reagents (Oxford Expression Technologies), recombinant viruses were harvested from the supernatant. The expression of M, N, GP5 and E from the recombinant baculoviruses were confirmed by Western blotting using HA tag specific monoclonal antibody (Sigma). Virus titers were determined by using the baculoQUANT ALL-IN-ONE™ Baculovirus DNA Extraction and Quantification Kit (Oxford Expression Technologies, Oxford, UK) following the manufacturer’s instructions.

### VLPs purification and characterization

TriEx SF9 cells were co-infected with recombinant baculoviruses containing PRRSV N, M, GP5, and E proteins at a MOI of 2 for N, GP5, and E proteins, and at a MOI of 3 for M protein with a starting cell concentration of 7.5 × 10^7^ cells/50 mL. Supernatants were harvested at 72 h post infection. Cellular debris was removed by centrifuging at 2000 rpm for 10 min in a Beckman Coulter Allegra 6 centrifuge. The supernatant was then filtered through a 0.22 μm filter and centrifuged at 28,000 rpm for 1 h in a SW28 Rotor Beckman Ultracentrifuge. Pellets were resuspended in PBS and subjected to a discontinuous 15–60% OptiPrep® density gradient (Sigma) centrifugation at 350,000 × g for 3 h in a Beckman OPTIMA 130 K Ultracentrifuge. The visible bands were collected and resuspended in PBS. Protein concentration was determined by using the Pierce® 660 nm protein assay reagent (Thermoscientific Inc.) following the manufacturer’s instructions.

For TEM study, the supernatant collected from the recombinant baculoviruses-infected TriEx SF9 cells were added to TSE Buffer containing 10 mM Tris HCl, 1 M EDTA, 100 mM NaCl with 20% sucrose. After centrifugation at 350,000 xg for 1.5 h 4 °C in a Beckman OPTIMA 130 K Ultracentrifuge, supernatant was discarded and the pellet was resuspended in 100 μL phosphate-buffered saline. Samples were processed for TEM study as described previously [28].

### Western blotting

TriEx Sf9 cells co-infected with recombinant baculoviruses containing PRRSV M, N, E, and GP5 gene were harvested at 72 h after infection. Cells were lysed with a lysis buffer containing 0.01 M Tris-HCl, 0.14 M NaCl, 0.025% NaN3, 1% Triton X-100, and protease/phosphatase inhibitors cocktail (Thermo Scientific, Rockford, IL). Cell lysates and purified VLPs were subjected to SDS-PAGE gel electrophoresis (Novex by Life Technologies, Carlsband, CA) and transferred to a nitrocellulose membrane (Life Technologies, Gaithersburg, MD). The membrane was then blocked for 1 h by rocking slowly at room temperature with 5% (w/v) milk powder in PBS plus 0.05% Tween 20 (PBST). Next, primary antibody, monoclonal mouse anti-HA antibody (Sigma-Aldrich, St. Louis, MO) diluted 1:5,000 in blocking buffer 5% (w/v) milk powder in PBST was added to the membrane and incubated overnight at 4 °C on the rocker. The secondary antibody, goat anti-mouse IRDye (LI-COR, Lincoln, NE) diluted 1:10,000 in PBST was added to the membrane and incubated for 1 h rotating at room temperature. Bands were visualized using the ODESSY Infrared Imaging System (LI-COR, Lincoln, NE).

### Animal immunization and challenge

Eighteen two-week-old piglets as determined to be PRRSV negative by the IDEXX PRRS X3 Ab ELISA were purchased from a local farm. After acclimation for a week, piglets were randomly divided into three groups. One group served as a control and received 2 ml of PBS intranasally. Another group received 250 μg of PRRSV VLP. The third group received 250 μg of PRRSV VLP plus 83.3 μg of 2′, 3′-cGAMP STING ligand (Invivogen). At two weeks after the primary immunization, a boost immunization of the same antigen preparation was administered intranasally. Two weeks after the boost immunization, all pigs were challenged with 2×10^5^ TCID_50_ of PRRSV-23983. Clinical signs were observed and recorded daily. At day 0, 3, 7, and 10 after challenge, serum and nasal swabs were collected. At day 10 after challenge, all pigs were necropsied and lung tissues were collected. Rectal temperatures were taken at day 0, 3, 7, and 10 after virus challenge.

### PRRSV specific antibody response

To measure the PRRSV N specific antibody response in the pigs, the serum samples were sent to the South Dakota State University Veterinary Diagnostic Laboratory for IDEXX PRRS X3 Ab ELISA. Data were presented as S/P ratios.

To measure PRRSV VLPs specific antibody response, the plates were coated overnight with the VLPs used in the vaccine at a concentration of 1 μg/ml in 50 μl of ELISA Coating Buffer (eBioscience, San Diego, CA.). The plates were washed 3 times with PBS containing 0.05% tween 20. The plates were then blocked with 100 μl of 5% skim milk in PBS for 1 h at room temperature. The plates were washed 5 times and the nasal samples in PBS were added at 100 μl in each well. The plates were incubated 1 h at room temperature. The plates were then washed 5 times and 100 μl of HRP conjugated anti-pig IgG or IgA was added to each well at a concentration of 1:10,000 for IgG and 1:100,000 for IgA. The plates are incubated at 37 °C for 1 h. Finally, the plates were washed 7 times and 100 μl of substrate solution (1× TMB Substrate, eBioscience) was added. The plates were incubated for 15 min in the dark at room temperature. The reaction was terminated with 50 μl of H_2_SO_4_ and read on a BioTek microplate reader at 450 nm.

### Cytokine response

Quantitative ELISA assays were used to determine the concentrations of IFN-α, IFN-γ, and IL-10 in the serum samples. The ELISA protocol for IFN-α has been described in detail previously [33]. Briefly, the recombinant porcine IFN-α (PBL Interferon Source) serially diluted 1:2 starting with 800 units/ml was used to generate the standard curve. One hundred microliters of diluted standards and 100 μl serum samples in duplicate were added to the plate.

The IL-10 and IFN-γ ELISA were performed using commercial ELISA kits (Thermo Fisher Scientific) by following the manufacturer’s instructions. One hundred microliters of standard was added to the appropriate wells at the appropriate dilutions in the standard diluent buffer. Fifty microliters of standard diluent buffer was added to each well containing 50 μl of the serum sample. The standard curve was used to determine the concentrations.

### Real-time RT-PCR

Real-time RT-PCR was used to determine the viral RNA copies in the serum samples. Viral RNA was extracted from 200 μl of serum by using the high pure viral nuclei acid kit (Roche-Applied-Science). cDNA synthesis was performed by using a High Capacity cDNA Synthesis Kit (Applied Biosystems Inc.) according to the manufacturer’s instructions. The forward primer (5′ GTC AAT CCA GAC CGC CTT TA 3′) and the reverse primer (5′ GAT CAG GCG CAC AGT ATG AT 3′) specific for the N gene of PRRSV were synthesized by the Integrated DNA Technologies. Real-time PCR was performed using the Brilliant II SYBR Green QRT-PCR Master Mix (Agilent Technologies, Santa Clara, CA) and the ABI 7500HT Real-Time Thermocycler (Applied Biosystems, Foster City, CA). Ct values were recorded.

### Statistical analysis

Student’s *t*-test was used to analyze significance. A *P*-value < 0.05 was considered significant.

## Results

### Generation and characterization of PRRSV VLP

**Fig. 1:**
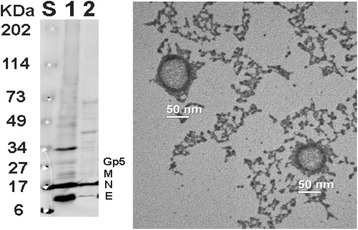
VLPs generated from recombinant baculoviruses expressing PRRSV proteins. *Left Panel*: Detection of PRRSV Gp5, M, N, and E proteins from recombinant baculoviruses-infected insect cells and from purified VLPs by Western blotting. Lane S: Protein standard; Lane 1: Cell lysates; Lane 2: Purified VLPs. *Right Panel*: TEM pictures of PRRSV VLPs

PRRSV VLP was generated from infection of insect TriEx™ Sf9 cells with recombinant baculoviruses expressing PRRSV M, N, E and Gp5 proteins. The expression of the viral proteins was detected in the cell lysates and purified VLP as shown in Fig. 1. Furthermore, the formation and presence of VLP in the supernatants of infected cells was verified by the VLP visualized under TEM. The size of VLP was approximately 50 nm (Fig. 1).

### Immune response in pigs

**Fig. 2:**
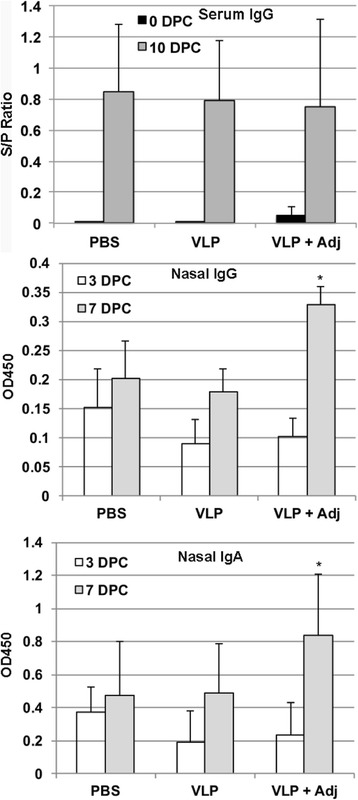
PRRSV N and VLPs specific antibody response. *Top Panel*: PRRSV N specific antibody response in the serum samples as detected by the IDEXX ELISA kit. *Middle Panel*: PRRSV VLPs specific IgG response in the nasal samples. *Lower Panel*: PRRSV VLPs specific IgA response in the nasal wash samples. Averages and standard deviations of 6 animals per group at 0 DPC, 3 DPC, and 10 DPC are shown. *indicates *p* < 0.05 between 3 DPC and 7 DPC samples of the VLPs with the adjuvant group

To examine the antibody response to VLP, we first used the IDEXX ELISA to detect the N protein specific antibody since the VLP does contain N protein. As shown in Fig. 2, we did not detect any significant difference in the antibody response before or after virus challenge, although a higher S/P ratio was observed prior to challenge in the VLP plus adjuvant group compared to the PBS and VLP groups. We used purified VLP as antigen to detect if any VLP specific antibody response was induced in the animals. We observed a significant increase in the OD reading for the VLP plus adjuvant group at day 7 after virus challenge when compared to day 3 after virus challenge, suggesting a boosting response elicited in the VLP plus adjuvant group for both IgG and IgA in the nasal samples. In contrast, no significant differences were observed for the PBS and VLP groups.

**Fig. 3:**
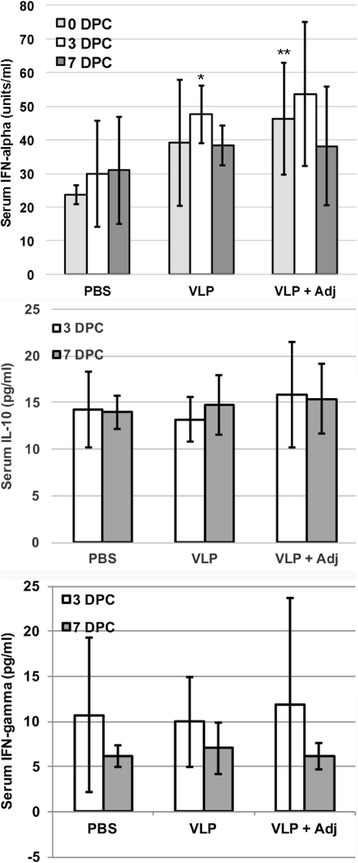
Cytokines in the serum of immunized and challenged animals. *Top Panel*: Interferon-α concentrations in the serum samples. *Middle Panel*: IL-10 concentrations in the serum samples. *Lower Panel*: Interferon-γ concentrations in the serum samples. Averages and standard deviations of 6 animals per group run in duplicate are shown. *indicates *p* < 0.05 between VLPs and PBS groups. **indicates *p* < 0.05 between VLPs + Adj and PBS groups

To examine the cellular response after virus challenge, we used ELISA to measure the interferon-γ in the serum. We found that all the animals showed a transient increase of IFN-γ at day 3 after virus challenge, followed by a decline at day 7 after virus challenge (Fig. 3). No significant differences between the vaccinated and the control groups were observed.

The adjuvant is known to induce type I interferon to enhance the immunogenicity of vaccine candidates. We examined the IFN-α in the serum before and after virus challenge. IFN-α was detected in all groups prior to virus challenge (Fig. 3). A significantly higher IFN-α response was seen in the VLP plus adjuvant group compared to the PBS group (*p* < 0.05) (Fig. 3). A transient increase in the IFN-α at day 3 after virus challenge was only observed in the VLP and VLP plus adjuvant group (Fig. 3). Although a slight increase in IFN-α was observed in the PBS group at day 3 and 7 after virus challenge, the level was mucher lower than the levels at day 0 after virus challenge in the VLP and VLP plus adjuvant groups. Furthermore, VLP induced a significantly higher IFN-α- than the PBS group at day 3 after virus challenge (*p* < 0.05). A higher IFN-α was also induced in the VLP plus adjuvant group at day 3 after virus challenge, but it is not significant when compared to PBS and VLP groups due to the high variability among the animals. The VLP plus adjuvant group did induce an overall higher IFN-α than the other two groups. This suggests that the VLP and VLP plus adjuvant delivered via the intranasal route have the capacity to activate type I interferon response in pigs.

PRRSV is known to induce IL-10 in infected animals. We examined the IL-10 in the serum using ELISA. We observed that no difference was found among the different vaccine groups in IL-10 response (Fig. 3). Compared to the transient increase of IFN-α and IFN-γ in the challenged animals, IL-10 appeared to be more stable and did not show any increase or decrease from day 3 to day 7 after virus challenge. This suggests that IL-10 induction is independent of VLP and adjuvant used.

### Rectal temperature, histological lesions, and viremia

**Fig. 4:**
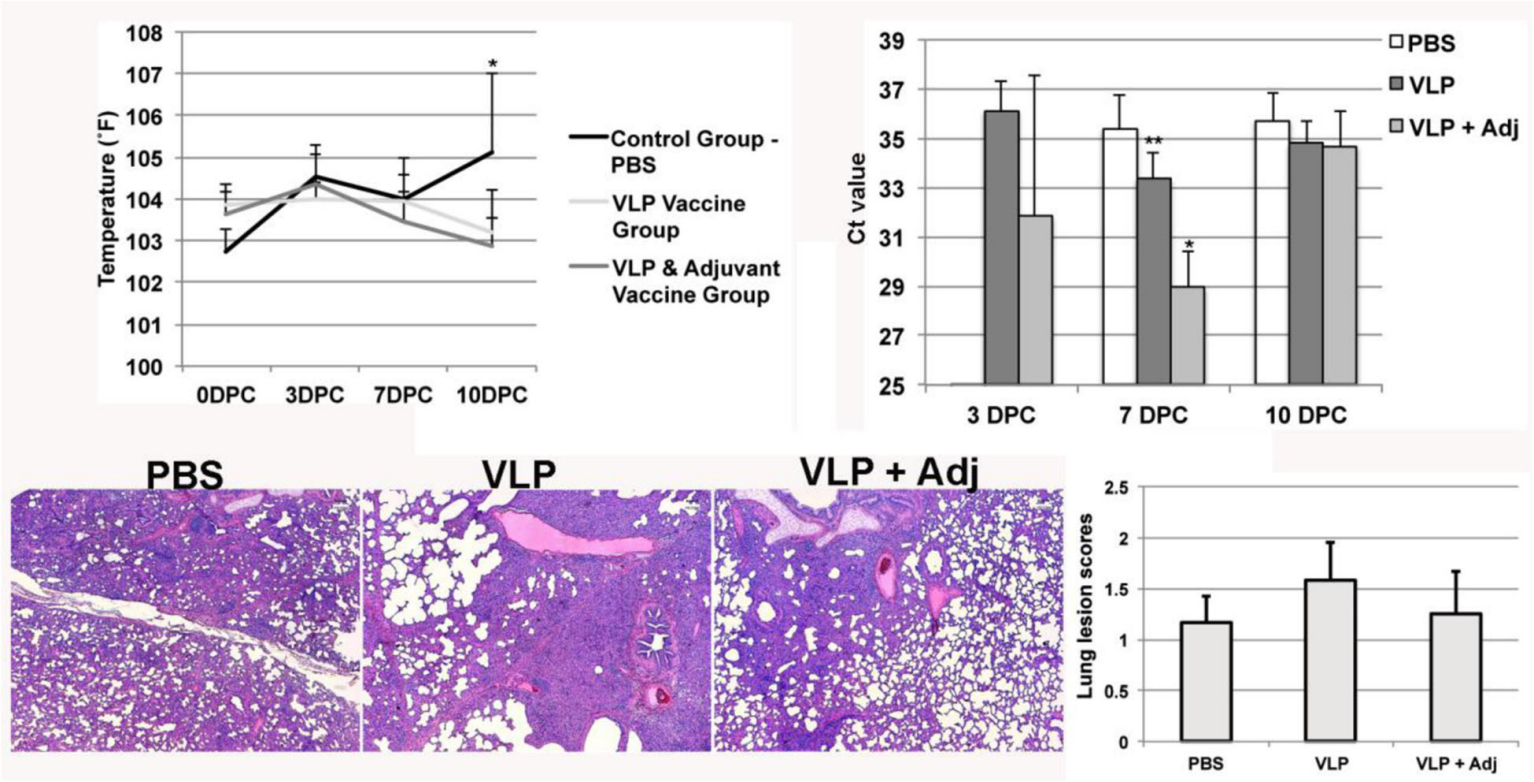
Rectal temperature, viremia, and histological lesions of lungs of animal challenged with PRRSV. *Top left panel*: Averages and standard deviations of rectal temperatures of 6 animals in each group at defined time points after virus challenge. * indicates *p* < 0.05 between PBS and VLPs & adjuvant group. *Top right panel*: Averages and standard deviations of Ct values of animals with detectable PRRSV RNA at duplicate qRT-PCR runs. *indicates *p* < 0.000001 between VLPs + Adjuvant group and PBS group. **indicates *p* < 0.01 between VLPs and PBS groups. *Lower left panel*: representative pictures showing the histological lesions of lungs at 10 DPC. Magnification 4×. *Lower right panel*: Averages and standard deviations of lung lesion scores of individual animals in each group. Scoring of the gross and microscopic lesions was based on the previously published data [11]. 0 = no lesions; 1 = mild interstitial pneumonia; 2 = moderate, multifocal interstitial pneumonia; 3 = moderate diffuse interstitial pneumonia; 4 = severe interstitial pneumonia

No clinical signs of PRRSV infection were observed in the vaccinated and challenged animals. However, the rectal temperature of animals in the PBS control group was significantly higher than the VLP plus adjuvant group at day 10 after virus challenge (Fig. 4). Similar histological lung lesions were observed in animals of all groups. The overall lesion scores in the PBS and the VLP plus adjuvant group appeared to be slightly milder than the VLP group (Fig. 4).

To quantify the viral RNA copies in the serum samples collected after virus challenge, we used qRT-PCR to detect the viral nucleocapsid gene transcript. We observed that none of the PBS vaccinated animals had detectable viral RNA at day 3 post challenge. While one out six animals in the VLP vaccine group and four out of six animals in the VLP plus adjuvant animals showed detectable viral RNA in the serum. At day 7 after virus challenge, 5 out of 6 animals in the PBS group demonstrated detectable viral RNA, while all 6 animals in the VLP and VLP plus adjuvant groups had detectable viral RNA. Furthermore, the viral RNA copies in the VLP and VLP plus adjuvant groups at day 7 after virus challenge were significantly higher than the PBS control group (Fig. 4). The VLP plus adjuvant group was also significantly higher than the VLP group. At day 10 after challenge, three out of six animals in the PBS group showed detectable viral RNA, while two out of six from the VLP group and four out of six animals from the VLP plus adjuvant group had detectable viral RNA. No significant difference was observed between the groups at day 10 after challenge. The results suggest that the VLP and the adjuvant exhibited a synergistic effect on enhancing PRRSV replication in pigs.

## Discussion

The genome of PRRSV contains 9 open-reading frames (ORFs). ORFs 2–7 encode viral structural proteins. Among the viral structural proteins, nucleocapsid protein (N, encoded by ORF 7), membrane protein (M, encoded by ORF 6), and glycoprotein 5 (GP5, encoded by ORF 5) are the most abundant structural proteins in the virions. These three proteins are essential to the formation of infectious virus particles for equine arterivirus, another member of the Arteriviridae [32]. Therefore, these three proteins likely play important roles in the assembly of virus particles for PRRSV, although other minor structural proteins including E (envelope protein), GP2, GP3, and GP4 (encoded by ORF 2, 3, and 4 respectively) may also contribute to the formation of infectious virus particles. A recent study has demonstrated a partial protection of PRRSV VLPs vaccine composed of five structural proteins including GP3, GP4, GP5, E, and M against homologous challenge when delivered together with PLGA nanoparticles [3]. In this study, we generated VLPs by expressing four PRRSV structural proteins including M, N, GP5, and E and assessed their immunogenicity and protective efficacy in pigs. No N protein and GP5 epitope specific antibody were detected in the serum at 2 weeks post boost, suggesting the VLPs dose and route of vaccination we used may not be sufficient in inducing an effective immune response. Alternatively, intramuscular injection could be a better choice of route of vaccination for VLPs. The reason we used intranasal delivery is because of recent studies showing cGAMP adjuvant as a mucosal adjuvant [4] and its superior performance than the intramuscular route when delivered together with antigens [15]. Nevertheless, we did observe a transient, but significant increase of VLPs specific IgG and IgA in the nasal samples of the VLPs plus adjuvant group at day 7 after challenge compared to day 3, but not in the PBS and VLPs groups. This result suggests that the adjuvant might be enhancing the immune response to VLPs. The significance of the VLPs specific antibody in protection remains to be determined. A higher viremia, but a lower rectal temperature, and milder histological lesions were observed in the VLPs plus the adjuvant group compared to the VLPs group. To our knowledge, this is first report on the potential use of a new class of STING ligand as an adjuvant for domestic animals including pigs. Previous studies mainly focus on its potency in inducing type I interferon in mice to enhance the immunogenicity of vaccine candidates including VLPs [14, 15].

A previous study showed a discrepancy between PRRSV viremia and histological lesions and clinical signs under field conditions [8]. We observed a similar phenomenon in the vaccinated and challenged animals. There is no correlation between viremia and clinical signs and histological lesions. This raises the importance of screening clinical naïve pigs for virus shedding in the herds. The non-sympomatic animals may carry a relatively high viral load and contribute to the disease transmission and outbreaks.

Both VLPs and VLPs with the adjuvant enhanced the interferon-α response compared to the PBS control. One previous study showed that Ebola VLPs are capable of inducing type I interferon [2], which corroborate our findings here. The effect of the 2′, 3′ cGAMP adjuvant in triggering the induction of type 1 interferon has been well documented in other animal models [9, 25]. Our data provide evidence for its effect in type I interferon induction in pigs. We speculate that the activation of innate immune response by VLPs and the adjuvant may facilitate PRRSV replication which leads to enhanced viremia in animals from these two groups when compared to the control group. A recent study has indeed shown that influenza virus replication is promoted by the activation of toll-like receptor 7 (TLR7) and RIG-I activation in the respiratory tract [21]. This could be a common phenomenon for many mucosal related virus infections. Brockmeier et al. reported the role of type I interferon in enhancing the PRRSV specific interferon-γ response in pigs [5]. In our study, a transient increase in interferon-γ at day 3 after challenge was observed in all groups. We did not see the enhancement of interferon-γ response by the higher interferon-α induction in the VLPs and VLPs plus adjuvant groups. This could be due to the short time periods we collected the samples after virus challenge. Our data support an earlier observation that PRRSV induces an early and transient interferon-γ activation [31]. Interestingly, IL-10 did not shown any change at day 3 and day 7 after virus challenge, suggesting that neither viremia nor interferon-α level affect IL-10 response in the animals.

Although we successfully generated PRRSV VLPs using the recombinant baculoviruses expressing Gp5, M, N and E proteins, we noticed the VLPs formation in the recombinant baculovirus co-infection model we used was not very efficient. This could be due to the protein combination we used is not ideal. Other minor structural proteins may be included in future studies to enhance the efficiency. Alternatively, strategies that can enhance the critical protein expression level should be considered to improve the VLPs formation efficiency as noted for human papillomavirus [23]. A multiBac expression system was proposed to enhance viral protein expression [23]. Contamination of baculovirus is another concern since it is difficult to remove all baculoviruses from purified VLPs. This has been described by others researchers [16]. We reason that the contaminating baculoviruses may serve as an additional trigger for type I interferon-α induction in VLPs vaccinated animals we observed. One previous study has indeed shown the effect of contaminating baculovirus in enhancing both innate and adaptive immune responses [16]. Another limitation of our study is that although Western blotting detected all four proteins from VLPs preparations, we could not be certain that all four proteins are actually incorporated into VLPs. Since all four proteins have the same HA tag, it would be difficult to differentiate them even by the immunogold TEM.

Overall, VLPs were generated from recombinant baculoviruses expressing Gp5, M, N and E proteins of PRRSV. The use of 2′, 3′-cGAMP adjuvant in the intranasal vaccination of PRRSV VLP enhanced virus replication, but not disease severity in pigs. A higher level of interferon-α production, but not interferon-γ and IL-10, is correlated with enhanced virus replication. Future studies should focus on incorporating other viral proteins of PRRSV into VLPs and improving VLPs assembly efficiency. Additionally, different dose of VLPs, different adjuvant and alternative route of vaccination such as intramuscular injection should be explored in the future to fully assess the feasibility of such a vaccine platform for PRRSV control and prevention.

## Conclusions

Intranasal immunizations of pigs with PRRSV VLPs and VLPs plus the 2’, 3’-cGAMP VacciGrade^TM^ adjuvant exacerbates viremia. A higher level of interferon-α production is correlated with enhanced virus replication. PRRSV VLPs and PRRSV VLPs plus the adjuvant fail to provide protection against PRRSV challenge.

## Abbreviations

CGAMP: Cyclic GMP-AMP
PRRSV: Porcine reproductive and respiratory virus
VLP: Virus like particles
